# Clinical evaluation of a commercial culture-free targeted next-generation sequencing test for diagnosis of drug-resistant tuberculosis

**DOI:** 10.1128/spectrum.03035-25

**Published:** 2025-12-12

**Authors:** Paolo Miotto, Rebecca E. Colman, Andrea M. Cabibbe, Paola M. V. Rancoita, Federico Di Marco, Andres De la Rossa, Christine Hoogland, Swapna Uplekar, Sacha Laurent, Daniela M. Cirillo, Camilla Rodrigues, Priti Kambli, Nestani Tukvadze, Nino Maghradze, Shaheed V. Omar, Lavania Joseph, Anita Suresh, Timothy C. Rodwell

**Affiliations:** 1 Emerging Bacterial Pathogens Unit, Division of Immunology, Transplantation and Infectious Diseases, IRCCS San Raffaele Scientific Institute, Milan, Italy; 2 FIND 91635, Geneva, Switzerland; 3 Division of Pulmonary, Critical Care, Sleep Medicine, and Physiology, University of San Diego 7119, San Diego, California, USA; 4 University Centre for Statistics in the Biomedical Sciences (CUSSB), Vita-Salute San Raffaele University 18985, Milan, Italy; 5 Hinduja Hospital and Medical Research Centre 29537, Mumbai, India; 6 Swiss Tropical and Public Health Institute 30247, Allschwil, Switzerland; 7 University of Basel 27209, Basel, Switzerland; 8 National Center for Tuberculosis and Lung Diseases 420719, Tbilisi, Georgia; 9 Centre for Tuberculosis, National & WHO Supranational TB Reference Laboratory, National Institute for Communicable Diseases (a division of the National Health Laboratory Service) 70687, Johannesburg, South Africa; ICON plc, London, United Kingdom

**Keywords:** multicenter study, molecular diagnostics, targeted next-generation sequencing (tNGS), drug resistance, tuberculosis

## Abstract

Rapid molecular diagnostics have significantly improved access to tuberculosis (TB) drug resistance detection and reduced turnaround times. However, these tools remain limited in their capacity to generate comprehensive resistance profiles, particularly for newer and repurposed anti-TB drugs. Targeted next-generation sequencing (tNGS) offers a promising alternative, enabling a broader resolution of *Mycobacterium tuberculosis* genomes and faster reporting compared to phenotypic drug susceptibility testing (DST). We conducted a prospective, cross-sectional, multicenter evaluation of the DeepChek 13-Plex KB Drug Susceptibility Testing (ABL Diagnostics S.A., France) tNGS assay for direct detection of TB drug resistance from clinical sputum samples. The study was performed at three reference laboratories in India, South Africa, and Georgia between April 2021 and June 2022. Adults (≥18 years) with confirmed pulmonary TB were enrolled. Sensitivity and specificity were assessed for key anti-TB drugs against a composite reference standard combining phenotypic DST and whole-genome sequencing. Of 832 participants enrolled, 694 (83.4%) were included in the final analysis. Sequencing was successful in 75.6% of samples. Failure rates were higher in samples with low or very low Xpert MTB/RIF categories. The ABL tNGS workflow showed ≥95% sensitivity for rifampicin, isoniazid, and levofloxacin; 92%–93% for pyrazinamide and moxifloxacin; 88% for ethambutol; and 72%–82% for bedaquiline and clofazimine. Specificity was ≥95% for all drugs. The ABL tNGS workflow enables comprehensive resistance profiling, including for new and repurposed TB drugs. However, higher bacillary loads are required to provide a valid test compared to current class-based tNGS assays recommended by the WHO, thus requiring further improvements.

**IMPORTANCE:**

Drug-resistant tuberculosis (TB) threatens progress in global TB control, yet current molecular tests detect resistance to only a few drugs. Targeted next-generation sequencing (tNGS) can read many resistance-related genes at once, offering faster and broader results than conventional culture-based testing. We evaluated a commercial tNGS workflow (DeepChek 13-Plex KB, ABL Diagnostics) for direct detection of drug resistance in sputum samples from adults with pulmonary TB in India, South Africa, and Georgia. Among 832 participants, sequencing produced valid results for most samples with moderate or high bacterial loads. The assay accurately identified resistance to key drugs—including rifampicin, isoniazid, fluoroquinolones, and newer medicines such as bedaquiline and clofazimine—while maintaining high specificity. These findings show that tNGS can deliver comprehensive resistance profiles, supporting tailored treatment for people with drug-resistant TB. Further refinement in sample preparation may expand its use to specimens with lower bacterial counts.

**CLINICAL TRIALS:**

This study is registered with ClinicalTrials.gov as NCT04239326.

## INTRODUCTION

The introduction of rapid molecular assays that can be performed directly on clinical samples has significantly improved the detection of drug-resistance in *Mycobacterium tuberculosis* (Mtb) complex strains and reduced time-to-result compared to culture-based methods. Despite these advancements, rapid molecular assays face several limitations: (i) they can only target a limited range of genetic loci or regions associated with resistance; (ii) they are not ideal for the detection of phenotypic resistance conferred by a variety of different mutations distributed across large genomic regions; (iii) they have reduced specificity for resistance detection due to difficulty distinguishing between silent mutations and those conferring resistance; and (iv) they lack flexibility for quick updates to include newly identified resistance mutations (1–3).

Targeted next-generation sequencing (tNGS) offers a culture-free and comprehensive molecular alternative for diagnosing drug-resistant tuberculosis (DR-TB). These approaches enable high-throughput analysis with greater accuracy across a broader spectrum of TB drugs from a single clinical sample, compared to current World Health Organization (WHO)-recommended molecular assays. They also provide a much faster time-to-result than phenotypic drug susceptibility testing (DST) (4, 5). In 2024, the WHO endorsed the use of tNGS for diagnosing resistance to isoniazid (INH), fluoroquinolones (FQ; namely, moxifloxacin [MXF] and levofloxacin [LFX]), bedaquiline (BDQ), linezolid (LZD), clofazimine (CFZ), pyrazinamide (PZA), ethambutol (EMB), amikacin (AMK), and streptomycin (SM) in people with bacteriologically confirmed rifampicin (RIF)-resistant pulmonary TB disease (6). The commercial products that met at least partial performance criteria for the newly established tNGS diagnostic class were Deeplex Myc-TB (GenoScreen), AmPORE-TB (Oxford Nanopore Technologies), and TBseq (ShengTing Biotech).

The Deeplex Myc-TB and AmPORE-TB assays were evaluated in a large multicenter clinical study which also generated data from an additional tNGS assay, the DeepChek 13-Plex KB Drug Susceptibility Testing Assay (ABL Diagnostics S.A., France) (7). However, DeepChek sequencing was not completed in time to be included in the initial analysis nor publication of results. As large, multicenter evaluations of tNGS assays for drug-resistant TB remain rare, it is critical to fully leverage all available data sets generated through such studies. An independent evaluation of the ABL DeepChek assay provides important additional evidence to inform the expanding role of tNGS in global TB diagnostics.

The ABL DeepChek tNGS assay targets mutations associated with resistance to first-line, second-line, and newly introduced TB drugs by amplifying key genomic regions of Mtb directly from clinical specimens. In this study, we aimed to evaluate the ABL tNGS workflow by determining its diagnostic accuracy for the detection of drug resistance and assessing overall sample and drug-specific failure rates. Diagnostic performance was evaluated in bacteriologically confirmed pulmonary TB (PTB) patients compared to a composite reference standard including whole genome sequencing (WGS) and phenotypic DST (pDST), as well as in the subgroup of bacteriologically confirmed pulmonary RIF-R TB patients.

## MATERIALS AND METHODS

### Study population and sample collection

Study design is described in Colman et al. (7). Briefly, a prospective, cross-sectional, multicenter clinical diagnostic accuracy study was conducted at reference laboratories in countries representing high TB burden and diverse epidemiological settings (Hinduja Hospital and Medical Research Centre, Mumbai, India; National Center for Tuberculosis and Lung Diseases, Tbilisi, Georgia; and National Institute for Communicable Diseases, Johannesburg, South Africa) to evaluate commercial tNGS end-to-end workflows for diagnosing DR-TB. End-to-end workflow was defined as a “design locked product intended for commercial use that includes DNA extraction, targeted gene amplification, sequencing of amplicons, bioinformatic analysis, and a report of interpreted sequencing result classifying the sequence result as resistant or susceptible.” Accordingly, no separate evaluation of individual workflow components (e.g., DNA extraction/purification procedures) was undertaken. Site-specific procedures, including extraction methods, were recorded but not independently assessed for their effect on assay performance. DNA was extracted from sputum sediments prepared by decontaminating sputum samples with the NaLC-NaOH method, as per manufacturer instructions. Eligible participants were aged 18 years or older, with PTB confirmed by GeneXpert MTB/RIF or Ultra (Cepheid, Sunnyvale, CA, USA), and at risk for DR-TB or proven to have RIF-R TB. The outcomes of this study have been recently published (7).

### Assay protocol

The DeepChek 13-Plex KB Drug Susceptibility Testing (ABL Diagnostics S.A., France) is an end-to-end workflow for direct and comprehensive detection of TB drug resistance. The ABL tNGS workflow for the Seq&Treat clinical study comprised different ABL-validated DNA extraction protocols at the three different study sites: MagSi-Dx Pathogen kit (Matgivio, Nuth, the Netherlands) (site: Georgia), MagNA Pure Total NA Isolation kit (Roche, Basilea, Switzerland) (site: India), and the MagNA Pure LC DNA Isolation kit III (Roche, Basilea, Switzerland) (site: South Africa). The DeepCheck PCR reagent kit included a 13-plex amplicon mix and library preparation reagents with resulting libraries run on an Illumina iSeq 100 Sequencing System for *Mycobacterium* species identification, and detection of drug resistance to RIF, INH, FQ, PZA, EMB, SM, second-line injectable drugs (AMK, capreomycin [CAP], kanamycin [KAN]), ethionamide (ETH), BDQ, and CFZ. Sequence data were automatically processed through the proprietary BacterioChek-Tb 2.0 software and mutations were interpreted using the ABL software which incorporates the second edition of the WHO Mutation Catalogue (8) plus additional evidence. The overall workflow is summarized in (Fig. S1). Comparisons between DeepCheck 13-Plex main features and the WHO-recommended tNGS solutions are provided in Table S1.

For AMK, CAP, and KAN, incomplete data or inconsistencies potentially linked to pre-analytical factors (e.g., storage or supply conditions) across sites could not be reconciled; therefore, analyses related to second-line injectables were excluded from evaluation. In addition, ABL tNGS workflow does not target LZD, and thus, LZD is excluded from the analysis.

### Reference standards

For RIF, INH, MXF, LFX, PZA, and EMB, a composite reference standard (cDST) was applied, which included pDST on MGIT culture combined with WGS data performed on isolates using the Illumina MiSeq platform at Medgenome (Bangalore, India), Sequencing Core Facility in NICD (South Africa), and San Raffaele Scientific Institute (Milan, Italy) and interpreted according to the 2021 WHO Mutations Catalogue (7, 9). A microbiological reference standard (pDST on MGIT culture) was considered for BDQ and CFZ as no resistance mutations were available for these drugs in the 2021 WHO Mutation Catalogue. In addition, to allow for direct comparison of results with the WHO evaluation of tNGS, comparison to pDST reference standard alone was considered for INH, MXF, and LFX.

Critical concentrations used for pDST are reported in Table S2; composite reference standard definition is reported in Table S3.

Samples that met any of the following exclusion criteria during the study were excluded from the primary analyses of the ABL tNGS workflow accuracy: culture negative results, culture positive but no Mtb complex identification available, specimens with growth of bacteria other than Mtb complex only, no valid WGS result (failed WGS quality criteria), ABL tNGS workflow accuracy analysis: no valid result for ABL tNGS workflow or no ABL tNGS result available.

Sample failures were defined as samples for which ABL tNGS workflow failed to produce any drug resistance interpretation data for the entire sample, whereas drug failures were defined as samples with tNGS interpretation results that resulted in “indeterminate” for at least one target for a given drug. Descriptive statistics of the failures were reported as absolute and relative frequencies. The following ranges were considered in describing sample and drug failure rates: low (<5%), moderate (5% to 15%), and high (>15%).

### Statistical analysis

The ABL index test diagnostic evaluation consisted of estimation of sensitivity and specificity with respect to the reference standard defined for that drug. The analysis was performed considering two types of samples: (i) all samples and (ii) only samples which were RIF-R by composite DST (cDST) reference standard. Moreover, since data were coming from three countries (Georgia, India, South Africa), the overall sample failure estimation and the test diagnostic evaluation were performed by estimating a common value accounting for the heterogeneity among the countries.

Heterogeneity among countries was measured using the index *I*^2^ and its 95% confidence interval was computed by using the R package meta (10). The *I*² statistic represents the proportion of the total variability in the results that is due to actual differences between sites rather than random chance. As documented in the literature, values of *I*^2^ closer to or greater than 50% were interpreted as indicating substantial heterogeneity (11). Sample failure, sensitivity, and specificity were estimated from univariate logistic mixed-effects models, where random effects were specified to account for the heterogeneity among countries using the R package glmmADMB (12). The corresponding data were also analyzed by considering the contingency tables separately by country for the sake of better understanding the source of heterogeneity (see Supplementary tables). To ensure reasonable 95% confidence intervals (*CIs*) in different scenarios, sensitivity estimates were computed only in case of a minimum of 25 resistant samples, and specificity estimates were computed only in case of a minimum of 50 susceptible samples. For sensitivity estimates, 25 resistant samples were sufficient to ensure a 95% confidence interval (CI) width of approximately 0.34 for sensitivity = 0.7, 0.31 for sensitivity = 0.8, 0.22 for sensitivity = 0.9. Regarding specificity, 50 susceptible samples are sufficient to ensure a width of the 95% CI of approximately 0.22 for specificity = 0.8, 0.20 for specificity = 0.85, 0.17 for specificity = 0.9, 0.11 for specificity = 0.95. These thresholds were set to ensure that the 95% confidence intervals around the estimates would be narrow enough to allow reliable interpretation. Without adequate sample sizes, site-level estimates could be misleading due to wide confidence intervals and increased uncertainty. The following ranges were considered for interpreting the sensitivity: low (<70%), moderate (between 70% and 90%), and high (>90%). For the specificity, the following ranges were considered: low (<80%), moderate (between 80% to 95%), and high (>95%). All analyses were performed with R 4.3.1 (13).

## RESULTS

**Fig 1:**
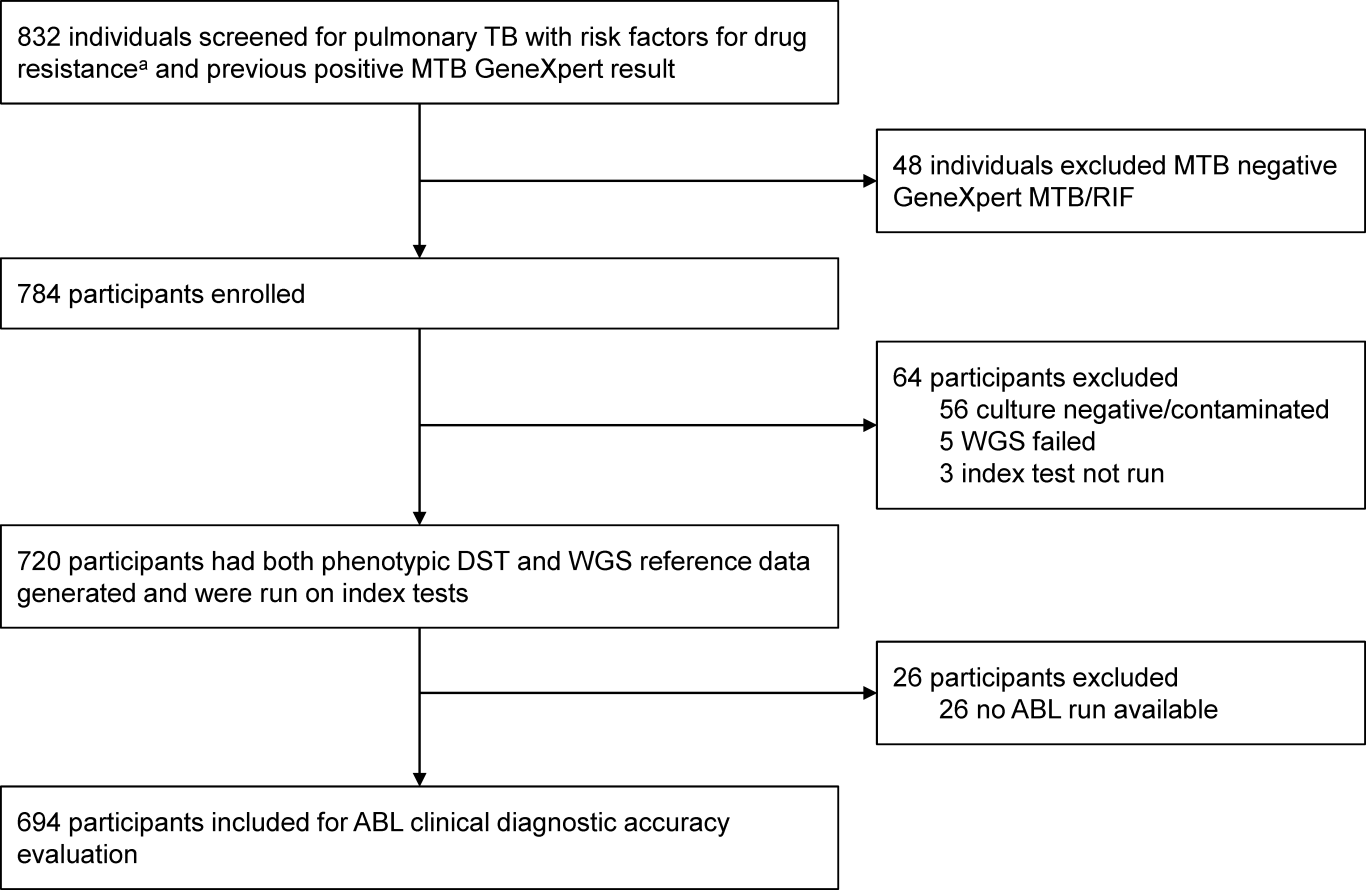
Participant flow chart. ^a^Risk factors for drug resistance were (i) a positive RIF-resistance result by Xpert MTB/RIF or Xpert MTB/RIF Ultra (i.e., “RIF resistance DETECTED”), or (ii) not responding to TB treatment with positive sputum smear or culture after ≥3 months of standard TB treatment, or (iii) previously diagnosed with RIF-R/MDR-TB and failed TB treatment with positive sputum smear or culture after ≥3 months of a standard MDR-TB regimen, or (iv) previously received >1 month of treatment for a prior TB episode, or (v) close contact with a known drug-resistant TB case.

Between 16 April 2021 and 30 June 2022, 832 people consented and met eligibility criteria for enrollment in the study across three study sites. Forty-eight people were excluded from the study due to MTB negative Xpert MTB/RIF results on the study sputum, leaving 784 patients who were enrolled and provided adequate Mtb positive sputa for study procedures. Of the 784 patients enrolled, 56 were excluded due to contaminated or negative cultures, 5 were excluded due to WGS sequencing failures, and 3 were excluded due to missing one or more tNGS index tests. The remaining 720 patients analyzed represented 91.8% of the total enrolled population. For the clinical diagnostic accuracy evaluation of the ABL tNGS workflow, an additional 26 participants were excluded due to the lack of ABL index test results, resulting in 694 patients included in the overall analysis, accounting for 83.4% of the total enrolled in the study (Fig. 1). The prevalence of phenotypic drug resistance for the original study ranged from 4.6% of clinical isolates with LZD resistance to 74.4% with resistance to INH (Table S4).

### Sample failure and drug-specific failure rate

Overall, the ABL tNGS workflow showed a moderate sequencing success with 75.6% (525/694) of clinical sediments producing at least partial drug-resistant profiles. The overall sample failure rate was 24.4% (169/694) (Table 1). More than 75% of failures were observed in the “medium” or lower Xpert categories (130 out of 169 failures) (Table 1; Fig. S2). As observed by Xpert semiquantitative category stratification, there was a trend of increasing failure rate as the category shifted from “high” (11.0%) to “very low” (86.7%) (Table 1). The *I*^2^ index showed a high heterogeneity of failure among the countries (98.4%, 95% CI = [97.1%; 99.1%]). After accounting for the heterogeneity, the estimated overall sample failure rate was 25% (95% CI [6.4%; 61.8%]). In contrast, drug-specific failure rates were generally low to moderate, ranging from 1% to 10%. The highest drug failure rates were observed for BDQ and CFZ, with each target failing in 69 samples (9.9%) (Table 1).

**TABLE 1:** ABL sample and drug failures, stratified by Xpert MTB/RIF semiquantitative result category^*a*^

	Xpert result category				
	High *n* (%)	Medium *n* (%)	Low *n* (%)	Very Low *n* (%)	Total
Sample failure	39 (11.0%)	53 (23.2%)	51 (62.2%)	26 (86.7%)	169 (24.4%)
RIF failure	2 (0.6%)	2 (0.9%)	2 (2.4%)	1 (3.3%)	7 (1.0%)
INH failure	9 (2.5%)	11 (4.8%)	6 (7.3%)	1 (3.3%)	27 (3.9%)
MXF failure	20 (5.6%)	17 (7.5%)	7 (8.5%)	3 (10.0%)	47 (6.8%)
LFX failure	20 (5.6%)	17 (7.5%)	7 (8.5%)	3 (10.0%)	47 (6.8%)
PZA failure	3 (0.8%)	2 (0.9%)	1 (1.2%)	0 (0.0%)	6 (0.9%)
BDQ failure	25 (7.1%)	30 (13.2%)	11 (13.4%)	3 (10.0%)	69 (9.9%)
CFZ failure	25 (7.1%)	30 (13.2%)	11 (13.4%)	3 (10.0%)	69 (9.9%)
EMB failure	7 (2.0%)	3 (1.3%)	2 (2.4%)	1 (3.3%)	13 (1.9%)
Total samples	354	228	82	30	694

^*a*^ This table reports the proportion of samples with complete assay failure (sample failures) and the proportion with drug-specific indeterminate results (drug failures) across categories of bacterial load as indicated by the Xpert MTB/RIF or MTB/RIF. Percentages were calculated using the number of evaluable samples within each category as the denominator. Sample failure = no sequence data interpretation produced; drug failure = no sequence data interpretation for specified drug. RIF: rifampicin; INH: isoniazid; MXF: moxifloxacin; LFX: levofloxacin; PZA: pyrazinamide; BDQ: bedaquiline; CFZ: clofazimine; EMB: ethambutol.

### ABL sensitivity and specificity analyses among people with bacteriologically confirmed PTB

The primary analyses focused on ABL tNGS diagnostic accuracy for the detection of resistance to RIF, INH, FQ (MXF, LFX), PZA, and EMB against a cDST reference and resistance to BDQ, CFZ against a pDST reference on the 525 samples producing tNGS sequencing results. The *I*^2^ index highlighted substantial heterogeneity across the sites for some of the drugs, especially for the estimation of the sensitivity (Table S5). Univariate mixed-effects models were used to estimate common sensitivity and specificity values; standard sensitivity and specificity values are also calculated for each site (Table 2). Across countries, ABL tNGS had ≥95% sensitivity for the detection of RIF, INH, and LFX, 92%–93% sensitivity for PZA and MFX, 88% sensitivity for EMB, and 72%–82% for BDQ and CFZ. Specificity was ≥95% for all drugs examined.

**TABLE 2:** Overall sensitivity and specificity of the ABL resistance detection in bacteriologically confirmed TB patients (*n* = 525)^*c*^

			Mixed-effects analysis	Mixed-effects analysis						Performance by site	Performance by site
Drug	Reference	n of drug failures (%)	Sensitivity [95% CI]	Specificity [95% CI]	Site	TP	FP	TN	FN	Sensitivity [95% CI]	Specificity [95% CI]
RIF	cDST	7 (1.4%)	99.1% [76.6%;100%]	99.0% [6.1%;100%]	Georgia India SAfrica	18 348 64	0 2 0	65 8 8	4 0 1	– 100% [99.0%;100%] 98.5% [91.7%;100%]	100% [94.5%;100%] – –
INH	cDST	27 (5.4%)	95.8% [78.7%;99.3%]	98.0% [90.8%;99.4%]	Georgia India SAfrica	26 343 34	0 1 1	44 12 25	2 2 8	92.9 [76.5%;99.1%] 99.4% [97.9%;99.9%] 81.0% [65.9%;91.4%]	– – –
MXF	cDST	47 (9.8%)	92.3% [71.0%;98.3%]	99.0% [96.2%;99.8%]	Georgia India SAfrica	5 248 10	0 1 1	52 102 50	2 5 2	− 98.0% [95.5%;99.4%] 83.3% [51.6%;97.9%]	100% [93.3%;100%] 99.0% [94.7%;100%] 98.0% [89.6%;100%]
LFX	cDST	47 (9.8%)	94.8% [78.9%;98.9%]	99.0% [96.2%;99.8%]	Georgia India SAfrica	5 248 10	0 1 1	53 103 50	1 4 2	– 98.4% [96.0%;99.6%] –	100% [93.2%;100%] 99.0% [94.8%;100%] 98.0% [89.6%;100%]
PZA	cDST	6 (1.2%)	93.3% [90.0%;95.6%]	96.0% [87.9%;98.9%]	Georgia India SAfrica	15 254 25	1 2 6	70 84 41	3 15 3	– 94.4% [91.0%;96.9%] 89.3% [71.8%;97.7%]	98.6% [92.4%;100%] 97.7% [91.9%;99.7%] –
BDQ^*a*^	pDST	69 (13.1%)	72.4% [53.7%;85.6%]	96.0% [84.7%;99.2%]	Georgia India SAfrica	0 12 9	0 31 2	50 289 54	2 2 4	– – –	100% [92.9%;100%] 90.3% [86.5%;93.3%] 96.4% [87.7%;99.6%]
CFZ^*a*^	pDST	69 (13.1%)	81.5% [62.5%;92.1%]	95.0% [85.1%;98.7%]	Georgia India SAfrica	0 14 8	0 29 3	51 289 56	1 2 2	– – –	100% [93.02%;100%] 90.9% [87.2%;93.8%] 94.9% [85.9%;98.9%]
EMB	cDST	13 (2.5%)	88.1% [84.3%;91.0%]	–^*b*^	Georgia India SAfrica	14 279 24	0 0 0	61 43 48	4 36 3	– 88.6% [84.5%;91.9%] 88.9% [70.8%;97.7%]	100% [94.1%;100%] – –

^*a*^ 1 sample failed for pDST.

^*b*^ –, not estimated since all pDST susceptible samples are tNGS susceptible, thus specificity is 100%.

^*c*^ Performance was assessed using specimens tested directly from decontaminated sputum sediment. Results are shown overall and stratified by site. Estimates account for inter-site heterogeneity using mixed-effects modeling. Sensitivity and specificity are expressed as percentages with 95% confidence intervals. Sensitivity by site was calculated only when ≥ 25 resistant samples were available, and specificity by site only when ≥ 50 susceptible samples were available, to ensure reasonably narrow 95% confidence intervals for reliable interpretation (see Materials and Methods for further details).

### ABL sensitivity and specificity analyses among people with bacteriologically-confirmed RIF-R PTB

The ABL tNGS workflow performance was also evaluated in RIF-R samples (defined by composite reference). For this subgroup, the *I*^2^ index highlighted substantial heterogeneity across the sites for some drugs (Table S6). Univariate mixed-effects models were used to estimate common sensitivity and specificity values (Table 3). Among RIF-R samples, the ABL tNGS had moderate (75%–90%) to high (>90%) sensitivity for all drugs and moderate specificity (with values between 91% and 95%) for the investigated drugs except for MXF, LFX, and EMB which had high specificity (≥99%).

**TABLE 3:** Overall sensitivity and specificity of the ABL resistance detection in RIF-R patients (*n* = 439)^*c*^

			Mixed-effects analysis	Mixed-effects analysis						Performance by site	Performance by site
Drug	Reference	n of drug failures (%)	Sensitivity [95% CI]	Specificity [95% CI]	Site	TP	FP	TN	FN	Sensitivity [95% CI]	Specificity [95% CI]
INH	cDST	11 (2.6%)	98.1% [78.5%;99.9%]	92.0% [73.0%;98.0%]	Georgia India SAfrica	19 341 33	0 1 1	2 4 17	0 2 8	− 99.4% [97.9%;99.9%] 80.5% [65.1%;91.2%]	– – –
MXF	cDST	23 (5.5%)	96.6% [85.4%;99.3%]	99.0% [94.8%;99.7%]	Georgia India SAfrica	4 248 10	0 1 1	10 93 43	0 4 2	– 98.4% [96.0%;99.6%] –	– 98.9% [94.2%;100%] –
LFX	cDST	23 (5.5%)	97.5% [83.6%;99.7%]	99.0% [94.8%;99.7%]	Georgia India SAfrica	4 248 10	0 1 1	10 95 43	0 2 2	– 99.2% [97.1%;99.9%] –	− 99.0% [94.3%;100%] –
PZA	cDST	6 (1.4%)	93.9% [90.7%;96.1%]	92.0% [79.5%;97.4%]	Georgia India SAfrica	14 254 25	1 2 6	6 74 32	1 15 3	– 94.4% [91.0%;96.9%] 89.3% [71.8%;97.7%]	− 97.4% [90.8%;99.7%] –
BDQ	pDST	40 (9.1%)	75.0% [55.1%;89.3%]	91.1% [87.7%;93.8%]	Georgia India SAfrica	0 12 9	0 31 2	12 279 47	1 2 4	– – –	− 90.0% [86.1%;93.1%] –
CFZ	pDST	40 (9.1%)	84.6% [65.1%;95.6%^*a*^]	91.4% [88.1%;94.1%]	Georgia India SAfrica	0 14 8	0 29 3	13 279 49	0 2 2	– 87.5% [61.7%;98.5%] –	− 90.6% [86.8%;93.6%] 94.2% [84.1%;98.8%]
EMB	cDST	5 (1.2%)	88.6% [84.8%;91.5%]	–^*b*^	Georgia India SAfrica	14 279 24	0 0 0	4 33 39	2 36 3	− 88.6% [84.5%;91.9%] 88.9% [70.8%;97.7%]	– – –

^*a*^ Estimated without Georgia since no pDST resistance sample is present in the data.

^*b*^ –, not estimated since all cDST susceptible samples are tNGS susceptible thus specificity is 100%.

^*c*^ Analysis was performed on decontaminated sputum sediment specimens. RIF-R was defined using composite reference standard. Results are shown overall and stratified by site. Estimates account for inter-site heterogeneity using mixed-effects modeling. Sensitivity and specificity are expressed as percentages with 95% confidence intervals. Sensitivity and specificity are expressed as percentages with 95% confidence intervals. Sensitivity by site was calculated only when ≥ 25 resistant samples were available, and specificity by site only when ≥ 50 susceptible samples were available, to ensure reasonably narrow 95% confidence intervals for reliable interpretation (see Materials and Methods for further details).

## DISCUSSION

Multiple commercial targeted NGS workflows are in development for TB diagnostics (14), including the commercial ABL tNGS assay for diagnosing DR-TB. This multicenter study, conducted across three countries, was designed to evaluate the performance of several commercial tNGS assays and included an evaluation of the ABL tNGS workflow. However, the ABL results were not included in the main analysis of tNGS workflows (7) due to delays in completing the ABL portion of work, and the results are presented here for the first time. As this evaluation focused on the assay as a design-locked, end-to-end workflow, we did not perform targeted investigations into the contribution of specific workflow steps to overall performance. The ABL tNGS workflow used three extraction protocols upfront of an Illumina iSeq100 platform and BacterioChek-Tb 2.0 software for genotypic detection of drug resistance directly from clinical sputum samples in patients with PTB. It is important to note that the study was not designed to achieve a prespecified accuracy for the evaluation of the new and repurposed TB drugs. The prevalence of phenotypic resistance to these drugs is low (~5%) in the community, and this limited the accuracy analysis for these drugs. The overall prevalence of phenotypic drug resistance (Table S4), clinical characteristics (Table S7), and Xpert results category distribution (Table S7) remained similar to those from the original evaluation study (7).

The evaluated ABL tNGS workflow showed moderate sequencing success with an estimated sample failure rate of 25% overall. When sample failures were stratified by Xpert MTB/RIF semiquantitative result category, ≈80% of the sample sequencing failures occurred in the samples with “medium” or lower Xpert semiquantitative categories (23.08% high, 31.36% medium, 30.18% low, 15.4% very low). Notably, more than 30% of failures were observed in the “medium” category (53/169 failures). These findings indicate that ABL tNGS sample failure rate is primarily driven by bacterial load. Therefore, in routine practice, ABL tNGS workflow could be implemented as a reflex test following an initial molecular diagnostic such as Xpert MTB/RIF, using the semi-quantitative category to guide feasibility. Specimens with medium or high bacillary load are most likely to yield interpretable results, whereas those with low or very low load have a higher risk of ABL tNGS failure. Since different DNA extraction/purification procedures were used across testing sites, and since the distribution of samples across Xpert semiquantitative categories varied by site, it was not possible to disentangle the relative contributions of each variable to the observed failure rates (Fig. S3).

Further research to assess different extraction systems and protocols in the ABL workflows and the resulting ABL tNGS performance would be useful to determine an optimal tNGS end-to-end workflow for this DR-TB assay.

According to the WHO target product profiles, minimal requirement for indeterminate results during DST is <10% (15). The ABL workflow showed lower drug target failure rates, with an average of 4.1% and a median of 2.9% across targets. Overall, drug-specific failure rates ranged from 1% to 10%, suggesting that when amplification is successful, the assay provides consistent and adequate coverage of resistance-associated genomic regions. The observed failure rates between the different anti-TB drugs were similar to those observed for AmPORE-TB, where overall, per drug target failures ranged from 0.7% to 11.8% of samples, with the PZA target having the highest failure rate (7). As the study was designed to evaluate the performance of the complete end-to-end workflow, we did not undertake targeted investigations to determine the underlying causes of these differences, such as amplicon design, GC content, or other technical factors.

Since substantial heterogeneity across sites was observed, we used univariate mixed-effects models to estimate overall sensitivity and specificity values. Heterogeneity should be interpreted with caution since it was derived by considering a small number of clustered groups (in this case, represented by the countries) (16). Consequently, sensitivity and specificity estimates by the mixed-effect models showed wide confidence intervals since only three heterogeneous sites were present.

**Fig 2:**
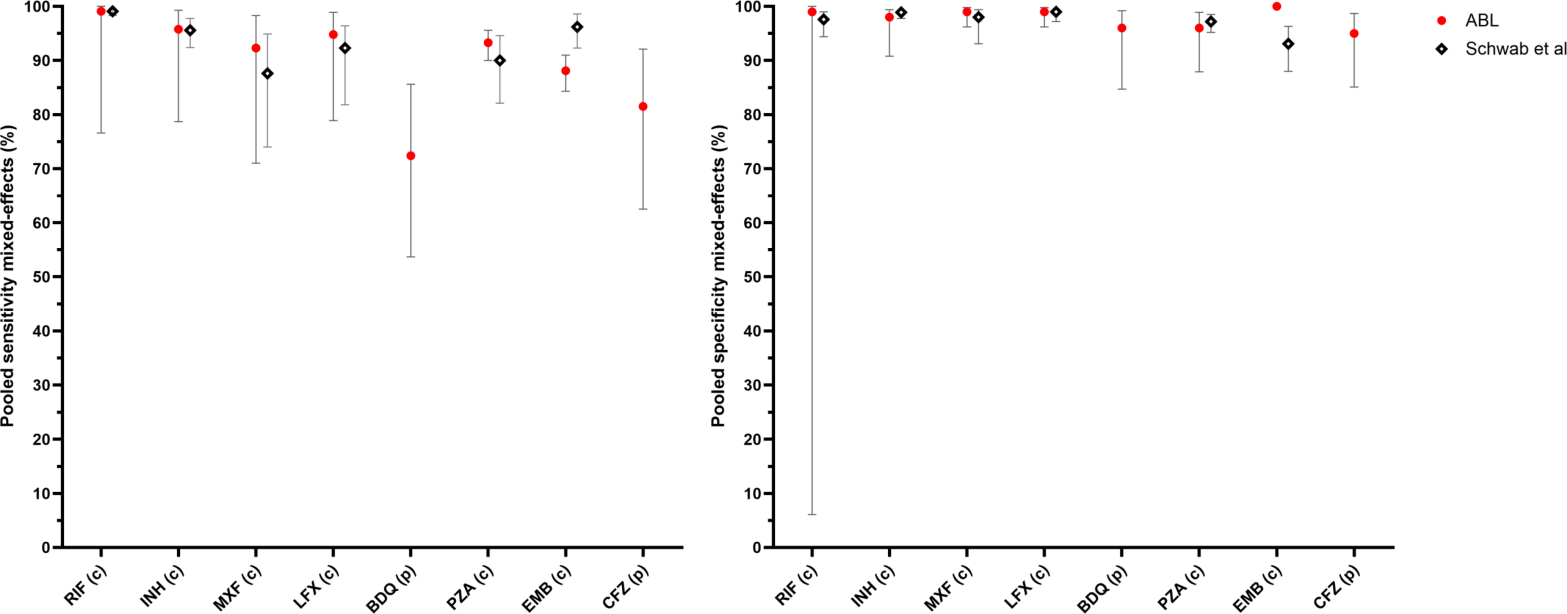
Sensitivity and specificity of the ABL workflow on the bacteriologically confirmed TB population. Estimates account for inter-site heterogeneity using mixed-effects modeling. Error bars indicate 95% confidence intervals. (P): phenotypic DST; (C): composite DST. Comparison with tNGS pooled performance is provided for the drugs considered on all samples from Schwab et al. (4).

**Fig 3:**
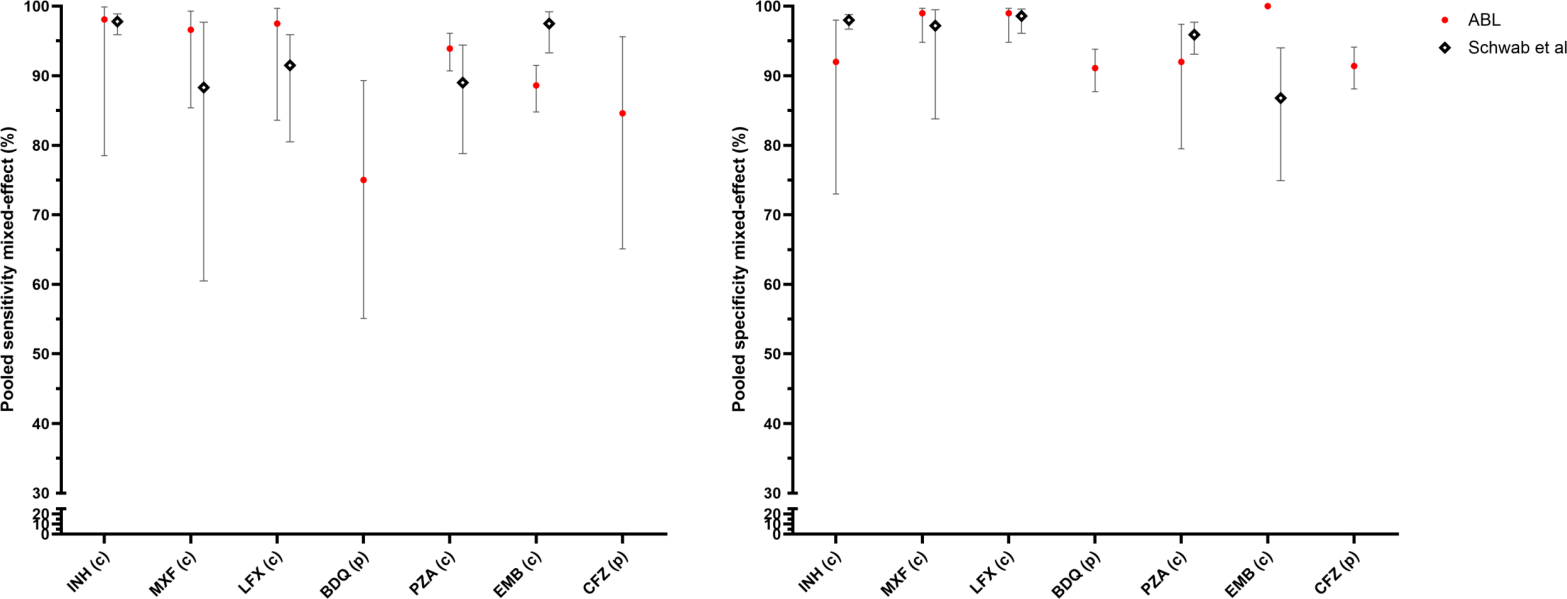
Sensitivity and specificity of the ABL workflow on bacteriologically confirmed RIF-R TB population. Estimates account for inter-site heterogeneity using mixed-effects modeling. Error bars indicate 95% confidence intervals. (P): phenotypic DST; (C): composite DST. LZD not targeted; AMK not considered. Comparison with tNGS pooled performance is provided for the drugs considered on patients with drug-resistant TB from Schwab et al. (4).

While the differences in reference standards prevent a direct comparison, an approximate evaluation between performance for the ABL workflow and a recent systematic review of tNGS performance (4) remains possible. The performance of the ABL tNGS workflow based on bacteriologically confirmed TB patient samples was similar to the combined estimates for different tNGS workflows, except for a lower sensitivity for EMB (Fig. 2). When considering studies that specifically recruited patients who had DR-TB (4) as a proxy for comparison with the bacteriologically confirmed RIF-R population in our study, the ABL workflow showed slightly higher sensitivity for FQ and PZA, but lower sensitivity for EMB (Fig. 3).

As this analysis used primarily cDST as reference, for direct comparison of ABL performance with the class-based performance reported in the WHO policy on tNGS (which used a mix of cDST and pDST [6]), we reanalyzed the data with the reference standards to match those used in the WHO evaluation: pDST for INH, MXF, LFX, BDQ, CFZ; cDST for RIF, PZA, EMB. As previously noted, we used univariate mixed-effects models to account for heterogeneity across sites. The ABL workflow met class-based sensitivity performance criteria for all drugs evaluated (RIF, INH, FQ, PZA), except for EMB which had a confidence interval below that of WHO policy for sensitivity evaluations (88.1%, 95% *CIs* 84.3%;91.0%) (heterogeneity assessment: Table S8; mixed-effects models: Table S9; Fig. S4, panel A). The ABL workflow also showed slightly lower specificity (–1% to –2%) performance relative to class (heterogeneity assessment: Table S8; mixed-effects models: Table S9; Fig. S4, panel B). Note that the confidence intervals of ABL workflow overlapped with the WHO policy specificity performance confidence intervals for all drugs (Fig. S4, panel B).

Performance for the ABL workflow was also compared with class-based performance for individual drugs as reported in the WHO policy for bacteriologically confirmed RIF-R population. The ABL workflow met class-based sensitivity performance criteria for all the drugs evaluated (heterogeneity assessment: Table S10; mixed-effects models: Table S1; Fig. S4, panel C). Note that the confidence intervals of ABL workflow overlapped with the WHO policy sensitivity performance confidence intervals for all drugs except for EMB. For specificity, the ABL workflow showed lower performance (−2% for FQ to −9% for INH; heterogeneity assessment: Table S10; mixed-effects models: Table S11; Fig. S4, panel D) although it remained within the WHO policy specificity performance confidence intervals for all drugs except INH, BDQ, PZA, and CFZ. Of note, for BDQ, the confidence interval of ABL workflow was entirely below the WHO policy specificity performance range, while for INH, PZA, and CFZ, the confidence intervals overlapped those reported in the WHO policy.

Overall, the ABL end-to-end tNGS workflow demonstrated moderate sensitivity for detecting Mtb drug resistance from clinical sputum samples. However, specificity was lower than that observed in similar tNGS assays (6). To investigate this, we conducted a discrepant analysis of false positive results (Table S12), which identified several contributing factors. These included the relatively small number of samples—where even a few false positives had a large proportional impact on estimated performance—and the presence of low-frequency variants that were not confirmed by WGS based on cultured isolates (e.g., RIF: 2/2; INH: 1/3; PZA: 7/9). In some cases, discrepancies reflect differences between the mutation-resistance interpretation catalogs (e.g., BDQ: 24/33, variants not interpreted by the WGS reference standard at the time of the analysis), or both tNGS and WGS identified variants that were interpreted as discordant with pDST (e.g., INH: 1/3; MXF: 8/10). These findings suggest that the reduced specificity may be partially addressed by refining the variant calling thresholds or interpretation rules used by the assay.

The study has several limitations. As explained in Colman et al. (7), the study was conducted during the COVID-19 pandemic, which changed the dynamics of TB detection and affected the enrollment numbers at each site. Second, we observed wider confidence intervals when stratifying data by testing site and when examining the new and repurposed drugs due to small sample size in these categories. Moreover, the study was explicitly enriched for individuals at risk for, or proven to have, RIF-R TB; thus, the analysis of the bacteriologically confirmed population without RIF-R is inherently biased toward RIF-R population. Third, by focusing on the end-to-end workflow as defined in the Materials and Methods section, this accuracy study did not explore additional aspects of the tNGS workflows. In particular, the study was not designed to evaluate the impact of DNA extraction or purification procedures on assay performance. Consequently, we were unable to investigate the underlying causes of the different drug failure rates observed. Finally, our study did not evaluate the added value of successful tNGS testing on direct specimens in cases where TB cultures were negative or contaminated, and thus neither pDST nor WGS was possible.

The ABL tNGS workflow is capable of detecting mutations associated with resistance to critical new and repurposed drugs. However, higher bacillary loads (i.e., mostly starting from medium Xpert category) are required to provide a valid test compared to current class-based tNGS assays recommended by the WHO, thus requiring further improvements in sample preparation and PCR-based enrichment. Data from this study were provided to the WHO Technical Advisory Group Meeting for the use of tNGS for the detection of DR-TB in January, 2025, and informed the following recommendation (17).

## Data Availability

Additional metadata and reference calls can be accessed via the Dryad Digital Repository at https://doi.org/10.5061/dryad.qjq2bvqs6. All the sequence data can be found on the NCBI Sequence Read Archive under accession number PRJNA1160005. Additional data are available upon request from the corresponding author. Templates of the informed consent forms may also be shared upon request.

## SUPPLEMENTAL MATERIAL

Supplemental material spectrum.03035-25-s0001.docx Tables S1 to S12; Fig. S1 to S4.

ASM does not own the copyrights to Supplemental Material that may be linked to, or accessed through, an article. The authors have granted ASM a non-exclusive, world-wide license to publish the Supplemental Material files. Please contact the corresponding author directly for reuse.
