# Supplementary material for: Clinical evaluation of a commercial culture-free targeted next-generation sequencing test for diagnosis of drug-resistant tuberculosis
Source: Microbiol Spectr. 2025 Dec 12;14(2):e03035-25. doi: 10.1128/spectrum.03035-25 (PMC12889153; doi:10.1128/spectrum.03035-25)
Supplement: Supplemental material — Tables S1 to S12; Fig. S1 to S4. [file spectrum.03035-25-s0001.docx]

**SUPPLEMENTARY MATERIAL**

**Table S1. Comparison between DeepChek 13-Plex and WHO-recommended tNGS solutions.** Summary of main features, and differences in technological approaches (1). RIF: rifampicin; INH: isoniazid; MXF: moxifloxacin; LFX: levofloxacin; PZA: pyrazinamide; BDQ: bedaquiline; LZD: linezolid; DLM: delamanid; CFZ: clofazimine; AMK: amikacin; EMB: ethambutol; SM: streptomycin; PAS: P-aminosalicyclic acid; ETH: ethionamide; DCS: D-cycloserine.

|  | **DeepChek 13-Plex (ABL)** | **Deeplex Myc-TB (GenoScreen)** | **Ampore-TB**  **(ONT)** | **TBSeq**  **(ShengTing)** |
| --- | --- | --- | --- | --- |
| **NGS technology** | Illumina | Illumina | Nanopore | Nanopore |
| **Species identification** | - | hsp65 | hsp65 | 16S, hsp65 |
| **Genotyping** | - | CRISPR/DR, phyloSNPs | CRISPR/DR | - |
| **RIF** | rpoB | rpoB | rpoB | rpoB |
| **INH** | fabG1, inhA, katG, furA-katG intergenic | ahpC, fabG1, katG, inhA | fabG1, katG, inhA | ahpC, katG, inhA |
| **MXF** | gyrA, gyrB | gyrA, gyrB | gyrA, gyrB | gyrA, gyrB |
| **LFX** | gyrA, gyrB | gyrA, gyrB | gyrA, gyrB | gyrA, gyrB |
| **PZA** | pncA | pncA | pncA | pncA |
| **BDQ** | Rv0678 | Rv0678 | Rv0678, atpE | Rv0678, atpE |
| **LZD** | - | rrl, rplC | rrl, rplC | rplC |
| **DLM** | - | - | ddn, fgd1, fbiA, fbiB, fbiC | - |
| **CFZ** | Rv0678 | Rv0678 | Rv0678 | Rv0678, atpE |
| **AMK** | rrs, eis | rrs, eis | rrs, eis | rrs, eis |
| **EMB** | embB | embB | embA, embB | embA, embB |
| **SM** | rrs, rpsL | gid, rrs, rpsL | gid, rrs, rpsL | gid, rrs, rpsL |
| **PAS** | - | - | - | folC, thyA |
| **ETH** | inhA, fabG1 | ethA, inhA, fabG1 | ethA, inhA, fabG1 | ethA, ahpC, inhA |
| **DCS** | - | - | - | alr |

**Table S2. Phenotypic DST concentrations tested.**

| Drug | WGS mutations in WHO catalogue 1^st^ edition | pDST tested (CC) | Composite drug call |
| --- | --- | --- | --- |
| Rifampicin (RIF) | Yes | Yes (0.5 µg/mL) | Yes |
| Isoniazid (INH) | Yes | Yes (0.1 µg/mL) | Yes |
| Ethambutol (EMB) | Yes | Yes (5.0 µg/mL) | Yes |
| Pyrazinamide (PZA) | Yes | Yes (100 µg/mL) | Yes |
| Moxifloxacin (MXF) | Yes | Yes (0.25 µg/mL) | Yes |
| Levofloxacin (LFX) | Yes | Yes (1.0 µg/mL) | Yes |
| Bedaquiline (BDQ) | No | Yes (1.0 µg/mL) | No |
| Clofazimine (CFZ) | No | Yes (1.0 µg/mL) | No |

**Table S3. Composite reference standard definition.** Criteria used to classify resistant or susceptible results for each drug in the composite reference standard.

| WGS | MGIT DST | Composite reference standard |
| --- | --- | --- |
| R | R | R |
| R | S | R |
| S | R | R |
| S | S | S |
| R | Not available | R |
| S | Not available | S |
| Not available | R | R |
| Not available | S | S |

**Table S4. Prevalence of phenotypic resistance for study.** ^a^from (2).

|  | pDST (study population^a^, n = 720) | pDST (ABL, n = 694) |
| --- | --- | --- |
| Drug | Total Resistant  N (%) | Total Resistant N (%) |
| RIF | 532 (73.9%) | 503 (72.5%) |
| INH | 536 (74.4%) | 517 (74.5%) |
| EMB | 401 (55.7%) | 385 (55.5%) |
| PZA | 379 (52.6%) | 371 (53.5%) |
| MFX | 312 (43.3%) | 306 (44.1%) |
| LFX | 313 (43.5%) | 308 (44.4%) |
| BDQ | 42 (5.8%) | 40 (5.8%) |
| CFZ | 40 (5.6%) | 38 (5.5%) |

**Table S5. Heterogeneity assessment on ABL dataset among all bacteriologically confirmed PTB patients.** Substantial heterogeneity (I^2^ ≳50%) highlighted in **bold**. *not estimated since all cDST sensible samples are tNGS sensible, thus specificity is 100%.

| Drug | Reference | I^2^ for sensitivity estimation [95% CI] | I^2^ for specificity estimation [95% CI] |
| --- | --- | --- | --- |
| RIF | cDST | **62.5% [0%;89.3%]** | 0% [0%;89.6%] |
| INH | cDST | **90.4% [74.7%;96.4%]** | 0% [0%;89.6%] |
| MXF | cDST | **85.0% [55.7%;94.9%]** | 0% [0%;89.6%] |
| LFX | cDST | **79.4% [34.3%;93.5%]** | 0% [0%;89.6%] |
| BDQ | pDST | 0% [0%;89.6%] | 2.0% [0%;89.8%] |
| PZA | cDST | **47.4% [0%;84.6%]** | **73.7% [12.0%;92.1%]** |
| CFZ | pDST | 0% [0%;89.6%] | 0% [0%;89.6%] |
| EMB | cDST | 0% [0%;89.6%] | -* |

**Table S6. Heterogeneity assessment on ABL dataset among bacteriologically-confirmed RIF-R PTB patients (defined by composite reference standard).** Substantial heterogeneity (I^2^ ≳50%) highlighted in **bold**. ^$^ estimated without Georgia, since no pDST resistance sample is present in the data. *not estimated since all cDST sensible samples are tNGS sensible, thus specificity is 100%.

| Drug | Reference | I^2^ for sensitivity estimation [95% CI] | I^2^ for specificity estimation [95% CI] |
| --- | --- | --- | --- |
| INH | cDST | **90.5% [74.9%;96.4%]** | 0% [0%;89.6%] |
| MXF | cDST | **73.1% [9.5%;92.0%]** | 0% [0%;89.6%] |
| LFX | cDST | **78.6% [31.3%;93.3%]** | 0% [0%;89.6%] |
| BDQ | pDST | 0% [0%;89.6%] | 0% [0%;89.6%] |
| PZA | cDST | 0% [0%;89.6%] | **63.0% [0%;89.4%]** |
| CFZ | pDST | -^$^ | 0% [0%;89.6%] |
| EMB | cDST | 0% [0%;89.6%] | -* |

**Table S7. Clinical characteristics of study participants.** ^a^from (1).

|  | Variable | Study population^a^ (n = 720), total (%) | ABL (n = 694), total (%) |
| --- | --- | --- | --- |
| HIV status | Positive  Negative  No record | 97 (13.5%)  533 (74.0%)  90 (12.5%) | 96 (13.8%)  511 (73.6%)  87 (12.5%) |
| AFB grade | +3  +2  +1  Scanty  Negative  No record | 132 (18.3%)  219 (30.4%)  172 (23.9%)  93 (12.9%)  103 (14.3)  1 (0.1%) | 132 (19.0%)  213 (30.7%)  166 (23.9%)  89 (12.8%)  93 (13.4%)  1 (0.1%) |
| Xpert semi quantitative result category | High  Medium  Low  Very low | 362 (50.3%)  238 (33.1%)  88 (12.2%)  32 (4.4%) | 354 (51.0%)  228 (32.9%)  82 (11.8%)  30 (4.3%) |

**Table S8. Heterogeneity assessment on ABL dataset among all bacteriologically confirmed PTB patients for comparison with WHO policy.** Substantial heterogeneity (I^2^ ≳50%) highlighted in **bold**. *not estimated since all pDST sensible samples are tNGS sensible, thus specificity is 100%.

| Drug | Reference | I^2^ for sensitivity estimation [95% CI] | I^2^ for specificity estimation [95% CI] |
| --- | --- | --- | --- |
| RIF | cDST | **62.5% [0%;89.3%]** | 0% [0%;89.6%] |
| INH | pDST | **90.6% [75.2%;96.4%]** | 0% [0%;89.6%] |
| MXF | pDST | **85.7% [58.2%;95.1%]** | 0% [0%;89.6%] |
| LFX | pDST | **66.3% [0%;90.3%]** | 0% [0%;89.6%] |
| BDQ | pDST | 0% [0%;89.6%] | 2.0% [0%;89.8%] |
| PZA | cDST | **47.4% [0%;84.6%]** | **73.7% [12.0%;92.1%]** |
| CFZ | pDST | 0% [0%;89.6%] | 0% [0%;89.6%] |
| EMB | cDST | 0% [0%;89.6%] | -* |

**Table S9. Overall sensitivity and specificity of the ABL resistance detection (performed direct on sediment, all bacteriologically confirmed TB patients, n = 525) compared to reference standard used in WHO policy.** Estimates account for inter‑site heterogeneity using mixed‑effects modelling. Sensitivity and specificity are expressed as percentages with 95% confidence intervals. Sensitivity by site was calculated only when ≥25 resistant samples were available, and specificity by site only when ≥50 susceptible samples were available, to ensure reasonably narrow 95% confidence intervals for reliable interpretation (See Methods for further details). ^a^ One sample failed for phenotypic DST. *not estimated since all pDST sensible samples are tNGS sensible, thus specificity is 100%.

|  |  |  | Mixed-effects analysis | Mixed-effects analysis |  |  |  |  |  | Performance by site | Performance by site |
| --- | --- | --- | --- | --- | --- | --- | --- | --- | --- | --- | --- |
| Drug | Reference | n. failures (%) | Sensitivity [95% CI] | Specificity [95% CI] | Site | TP | FP | TN | FN | Sensitivity [95% CI] | Specificity [95% CI] |
| RIF | cDST | 7 (1.3%) | 99.1% [76.6%;100%] | 99.0% [6.1%;100%] | Georgia  India  SAfrica | 18  348  64 | 0  2  0 | 65  8  8 | 4  0  1 | -  100% [99.0%;100%]  98.5% [91.7%;100%] | 100% [94.5%;100%]  -  - |
| INH | pDST | 27 (5.1%) | 95.8% [78.3%;99.3%] | 97% [87.2%;99.2%] | Georgia  India  SAfrica | 26  343  33 | 0  1  2 | 44  12  25 | 2  2  8 | 92.9 [76.5%;99.1%]  99.4% [97.9%;99.9%]  80.5% [65.1%;91.2%] |  |
| MXF | pDST | 47 (9%) | 91.6% [68.4%;98.2%] | 95.0% [89.4%;98.1%] | Georgia  India  SAfrica | 5  242  8 | 0  7  3 | 52  102  50 | 2  5  2 | -  98.0% [95.3%;99.3%]  80.0% [44.4%;97.5%] | 100% [93.2%;100%]  93.6% [87.2%;97.4%]  94.3% [84.3%;98.8%] |
| LFX^a^ | pDST | 47 (9%) | 96.6% [81.2%;99.5%] | 96.0% [92.6%;98.2%] | Georgia  India  SAfrica | 5  243  9 | 0  6  2 | 52  103  51 | 1  4  1 | -  98.4% [95.9%;99.6%]  - | 100% [93.2%;100%]  94.5% [88.4%;98.0%]  96.2% [87.0%;99.5%] |
| PZA | cDST | 6 (1.2%) | 93.3% [90.0%;95.6%] | 96.0% [87.9%;98.9%] | Georgia  India  SAfrica | 15  254  25 | 1  2  6 | 70  84  41 | 3  15  3 | -  94.4% [91.0%;96.9%]  89.3% [71.8%;97.7%] | 98.6% [92.4%;100%]  97.7% [91.9%;99.7%]  - |
| BDQ^a^ | pDST | 69 (13.1%) | 72.4% [53.7%;85.6%] | 96.0% [84.7%;99.2%] | Georgia  India  SAfrica | 0 12 9 | 0 31 2 | 50 289 54 | 2 2 4 | -  - - | 100% [92.9%;100%] 90.3% [86.5%;93.3%]  96.4% [87.7%;99.6%] |
| CFZ^a^ | pDST | 69 (13.1%) | 81.5% [62.5%;92.1%] | 95.0% [85.1%;98.7%] | Georgia  India  SAfrica | 0 14 8 | 0 29 3 | 51 289 56 | 1 2 2 | - - - | 100% [93.0%;100%] 90.9% [87.2%;93.8%]  94.9% [85.9%;98.9%] |
| EMB | cDST | 13 (2.5%) | 88.1% [84.3%;91.0%] | -* | Georgia  India  SAfrica | 14 279 24 | 0 0 0 | 62 43 48 | 4 36 3 | - 88.6% [84.5%;91.9%] 88.9% [70.8%;97.7%] | 100% [94.1%;100%]  - - |

**Table S10. Heterogeneity assessment on ABL dataset among RIF-R (defined by composite reference standard) PTB patients for comparison with WHO policy.** Substantial heterogeneity (I^2^ ≳50%) highlighted in **bold**. ^$^ estimated without Georgia, since no pDST resistance sample is present in the data. *not estimated since all pDST sensible samples are tNGS sensible, thus specificity is 100%.

| Drug | Reference | I^2^ for sensitivity estimation [95% CI] | I^2^ for specificity estimation [95% CI] |
| --- | --- | --- | --- |
| INH | pDST | **90.5% [74.9%;96.4%]** | 0% [0%;89.6%] |
| MXF | pDST | **76.2% [21.8%;92.7%]** | 0% [0%;89.6%] |
| LFX | pDST | **52.3% [0%;86.3%]** | 0% [0%;89.6%] |
| BDQ | pDST | 0% [0%;89.6%] | 0% [0%;89.6%] |
| PZA | cDST | 0% [0%;89.6%] | **63.0% [0%;89.4%]** |
| CFZ | pDST | -^$^ | 0% [0%;89.6%] |
| EMB | cDST | 0% [0%;89.6%] | -* |

**Table S11. Overall sensitivity and specificity of the ABL resistance detection (performed direct on sediment, RIF-R patients defined by composite reference, n = 439) compared to reference standard used in WHO policy.** Estimates account for inter‑site heterogeneity using mixed‑effects modelling. Sensitivity and specificity are expressed as percentages with 95% confidence intervals. Sensitivity by site was calculated only when ≥25 resistant samples were available, and specificity by site only when ≥50 susceptible samples were available, to ensure reasonably narrow 95% confidence intervals for reliable interpretation (See Methods for further details). ^a^ One sample failed for phenotypic DST. ^$^estimated without Georgia, since no pDST resistance sample is present in the data. *not estimated since all pDST sensible samples are tNGS sensible, thus specificity is 100%.

|  |  |  | Mixed-effects analysis | Mixed-effects analysis |  |  |  |  |  | Performance by site | Performance by site |
| --- | --- | --- | --- | --- | --- | --- | --- | --- | --- | --- | --- |
| Drug | Reference | n. failures (%) | Sensitivity [95% CI] | Specificity [95% CI] | Site | TP | FP | TN | FN | Sensitivity [95% CI] | Specificity [95% CI] |
| INH | pDST | 11 (2.5%) | 98.1% [78.5%;99.9%] | 92% [73.0%;98%] | Georgia  India  SAfrica | 19  341  33 | 0  1  1 | 2  4  17 | 0  2  8 | -  99.4% [97.9%;99.9%]  80.5% [65.1%;91.2%] | - - - |
| MXF | pDST | 23 (5.2%) | 96.3% [82.8%;99.3%] | 94% [88.5%;96.5%] | Georgia  India  SAfrica | 4  242  8 | 0  7  3 | 10  93  43 | 0  4  2 | -  98.4% [95.9%;99.6%]  - | - 93% [86.1%;97.1%]  - |
| LFX^a^ | pDST | 23 (5.2%) | 98.8% [96.5%;99.6%] | 95% [90.1%;97.4%] | Georgia  India  SAfrica | 4  243  9 | 0  6  2 | 10  95  44 | 0  2  1 | -  99.2% [97.0%;99.9%]  - | -  94.1% [87.5%;97.8%]  - |
| PZA | cDST | 6 (1.4%) | 93.9% [90.7%;96.1%] | 92% [79.5%;97.4%] | Georgia  India  SAfrica | 14  254  25 | 1  2  6 | 6  74  32 | 1  15  3 | -  94.4% [91.0%;96.9%]  89.3% [71.8%;97.7%] | -  97.4% [90.8%;99.7%]  - |
| BDQ^a^ | pDST | 40 (9.1%) | 75% [56.0%;87.6%] | 91% [87.8%;93.6%] | Georgia  India  SAfrica | 0 12 9 | 0 31 2 | 12 279 47 | 1 2 4 | -  -  - | -  90% [86.1%;93.1%]  - |
| CFZ^a^ | pDST | 40 (9.1%) | 84.6% [65.5%;94.1%]^$^ | 91% [88.1%;93.9%] | Georgia  India  SAfrica | 0  14  8 | 0  29  3 | 13  279  49 | 0  2  2 | -  -  - | -  90.6% [86.8%;93.6%]  94.2% [84.1%;98.8%] |
| EMB | cDST | 5 (1.2%) | 88.6% [84.8%;91.5%] | -* | Georgia  India  SAfrica | 14  279  24 | 0  0  0 | 4  33  39 | 2  36  3 | -  88.6% [84.5%;91.9%]  88.9% [70.8%;97.7%] | - - - |

**Table S12. Details of discrepancies affecting specificity (false positive samples) and sensitivity (false negative samples) for the ABL tNGS assay.** Includes mutation profiles and reference standard comparison. 1-15% low-frequency variants; 15-40% subclonal mutations, possible heterogeneity; Geno-pheno discordance: tNGS and WGS concordance; pDST discordance; True discordance: pDST and WGS concordance; tNGS discordance. *WGS not available

| **Drug** | **Reference** | **FALSE POSITIVES** | | | **FALSE NEGATIVES** | |
| --- | --- | --- | --- | --- | --- | --- |
|  |  | **N** | **tNGS variant call** | **Description** | **N** | **Description** |
| **RIF** | cDST | 1 | rpoB_S450* | low-frequency variants | 2 | Geno-pheno discordance |
|  |  | 1 | rpoB_T444P | Subclonal mutations, possible heterogeneity | 3 | True discordance |
| **INH** | pDST | 1 | katG_E208* | low-frequency variants | 11 | Geno-pheno discordance |
|  |  | 1 | fabG1_c-15 | Geno-pheno discordance | 1 | True discordance |
|  |  | 1 | fabG1_c-15t+katG_S315T | True discordance |  |  |
| **MFX** | pDST | 8 | gyrA_A90V (n 7); gyrB_D461N | Geno-pheno discordance | 8 | Geno-pheno discordance |
|  |  | 2 | gyrA_A90V; gyrB_D461N | True discordance | 1 | True discordance |
| **PZA** | cDST | 3 | pncA_T87A; pncA_S104N; pncA_K96N | low-frequency variants | 19 | Geno-pheno discordance |
|  |  | 4 | pncA_T142M; pncA_S66L; pncA_H71Y; pncA_D33Y+pncA_R148C | Subclonal mutations, possible heterogeneity | 2 | True discordance |
|  |  | 2 | pncA_131fs; pncA_G16D | True discordance |  |  |
| **BDQ*** | pDST | 23 | Rv0678_E104* (n 6); Rv0678_E81* (n 4); Rv0678_ E104*+Rv0678_E106*; Rv0678_47fs; Rv0678_E49* (n 4); Rv0678_E21*+Rv0678_E147*; Rv0678_E49*+Rv0678_E81*; Rv0678_E106* (n 3); Rv0678_E147*; Rv0678_E113* | low-frequency variants | 8 | Geno-pheno discordance |
|  |  | 1 | Rv0678_E106* | Subclonal mutations, possible heterogeneity |  |  |
|  |  | 9 | Rv0678_46fs (n 4); Rv0678_47fs (n 3); Rv0678_46fs+Rv0678_E49*; Rv0678_16fs | True discordance |  |  |

**Figure S1. Summary of the DeepChek Assay 13-Plex KB Drug Susceptibility Testing assay workflow.** The times indicated in the workflow are theoretical estimates and were not measured in this study.


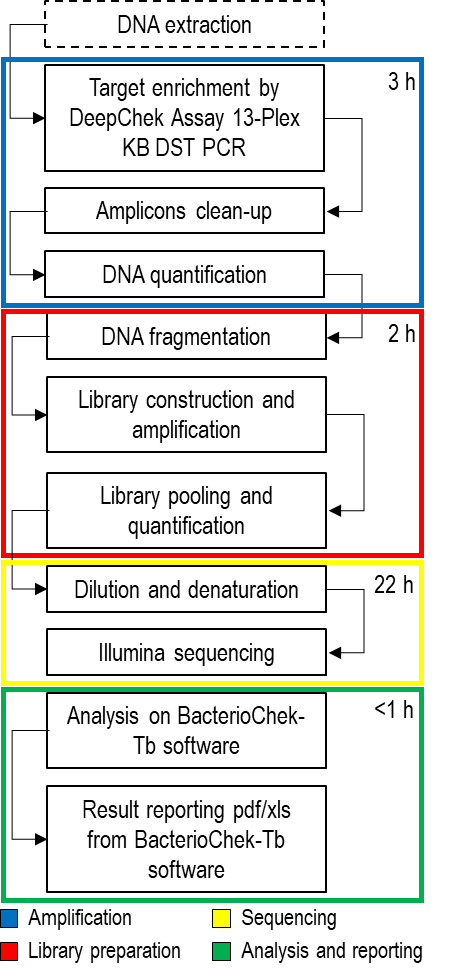


**Figure S2. ABL sample sequencing results stratified by Xpert MTB/RIF semiquantitative category**. (left) Stacked bars showing the proportion of samples with complete, partial, or failed results within each semiquantitative category. (right) Stacked bars showing the distribution of Xpert MTB/RIF semiquantitative categories among samples with complete assay failure. Raw numbers are also reported.


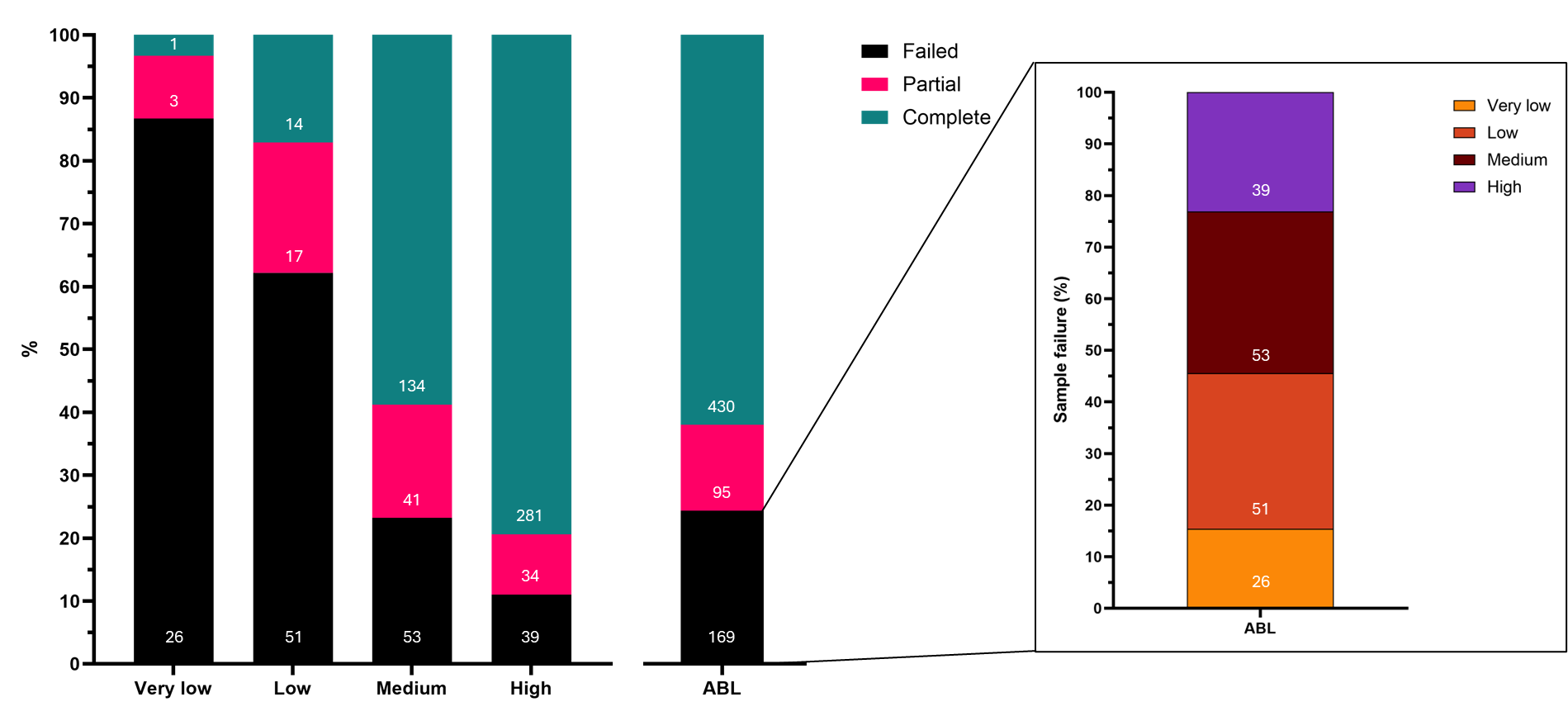


**Figure S3. Description of the cohort and sample failure rates by study site, stratified by Xpert MTB/RIF semiquantitative result category.** (left) Stacked bars showing the distribution of Xpert MTB/RIF semiquantitative categories across the three participating study sites. (middle) Stacked bars showing the proportion of each semiquantitative category among samples with complete assay failure at each site. Raw numbers are reported. (right) Details on the DNA extraction methods used at each site are also provided.


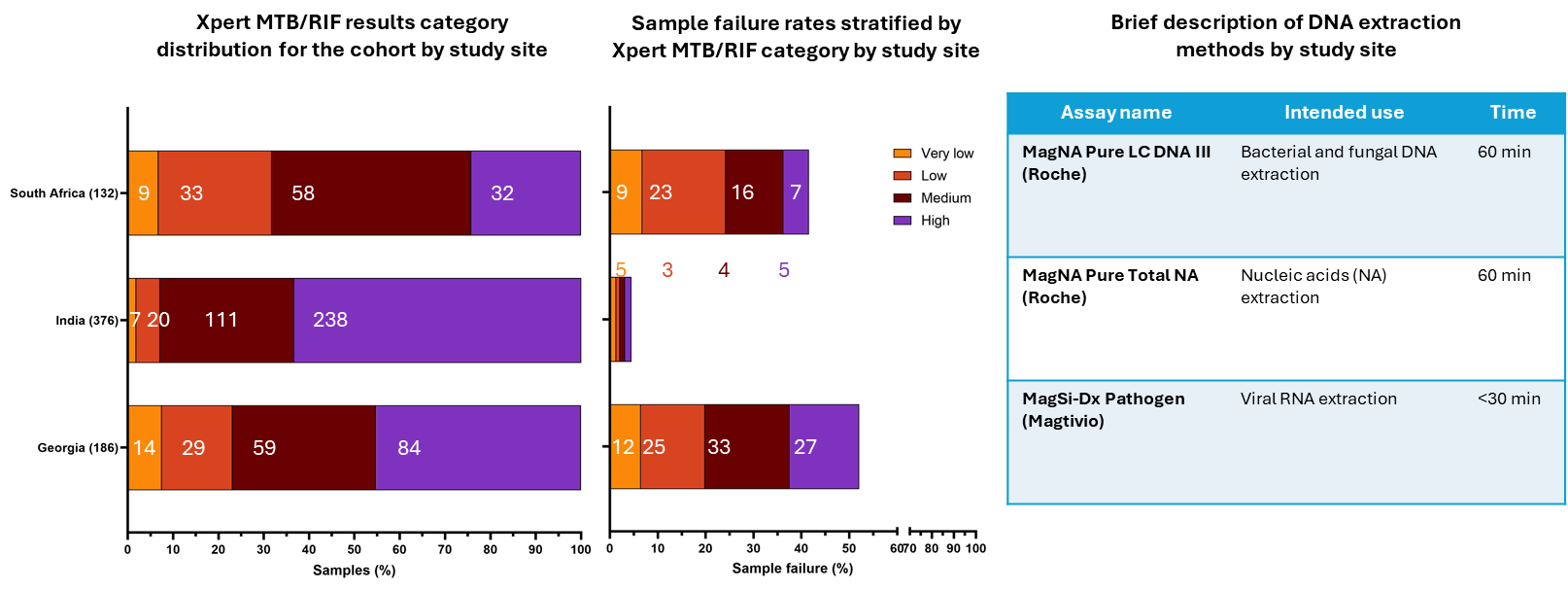


**Figure S4. Sensitivity (left) and specificity (right) of the ABL workflow (accounting for heterogeneity, mixed-effects) compared with the class-based performance of individual drugs as reported in the WHO policy.** Panels A–B: bacteriologically confirmed TB population; Panels C–D: RIF‑resistant TB population. (p): phenotypic DST; (c): composite DST.


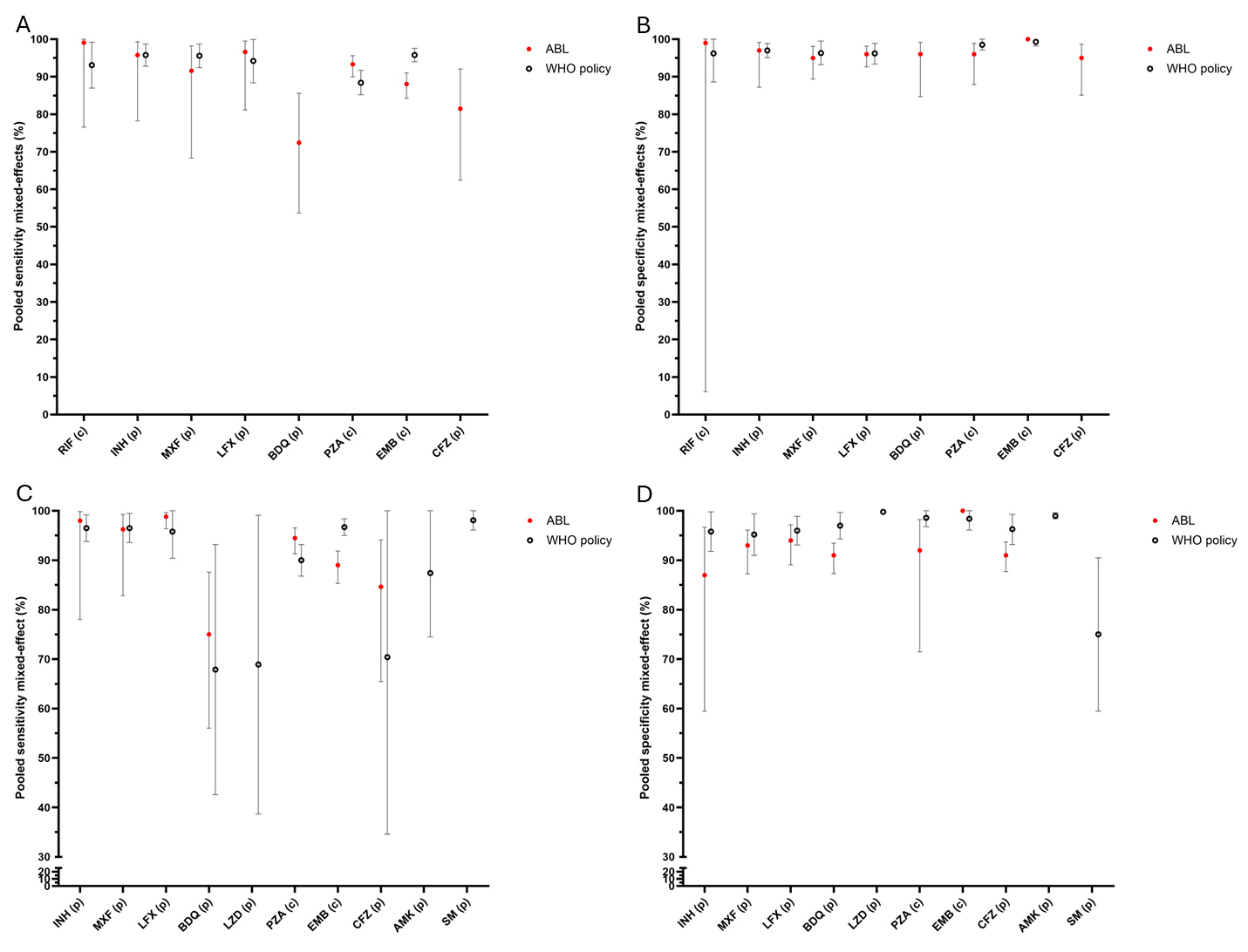


**REFERENCES**

1. Web Annex A. Information sheets. In: WHO operational handbook on tuberculosis. Module 3: diagnosis – rapid diagnostics for tuberculosis detection, third edition. Geneva: World Health Organization; 2024. Licence: CC BY-NC-SA 3.0 IGO.
2. Colman RE, Seifert M, De la Rossa A, Georghiou SB, Hoogland C, Uplekar S, Laurent S, Rodrigues C, Kambli P, Tukvadze N, Maghradze N, Omar SV, Joseph L, Suresh A, Rodwell TC. 2024. Evaluating culture-free targeted next-generation sequencing for diagnosing drug-resistant tuberculosis: a multicentre clinical study of two end-to-end commercial workflows. Lancet Infect Dis.
